# Follow-Up and Monitoring of Children Needing Long Term Home Ventilation

**DOI:** 10.3389/fped.2020.00330

**Published:** 2020-06-22

**Authors:** Sonia Khirani, Alessandro Amaddeo, Lucie Griffon, Agathe Lanzeray, Theo Teng, Brigitte Fauroux

**Affiliations:** ^1^ASV Santé, Gennevilliers, France; ^2^Pediatric Noninvasive Ventilation and Sleep Unit, AP-HP, Hôpital Necker-Enfants Malades, Paris, France; ^3^Université de Paris, VIFASOM, Paris, France

**Keywords:** noninvasive respiratory support, child, follow-up, monitoring, hospital, home, weaning

## Abstract

Once continuous positive airway pressure (CPAP) or noninvasive ventilation (NIV) is started in a child, and the child is discharged home, follow-up needs to be organized with regular visits in order to check the tolerance and efficacy of the treatment. But there is a lack of validated clinical guidelines, mainly because of the heterogeneity of the ventilator servicing, the costs and health care systems among countries. Therefore, visits timing and strategies to monitor CPAP/NIV are not clearly defined. Moreover, depending on various factors such as the underlying disorder, the medical stability, the age of the child, and socio-economic factors, follow-up usually ranges between 1 month and 3–6 months, or even 1 year following treatment initiation, with an overnight hospital stay, an out-patient visit, a home visit, via telemonitoring or telemedicine, alone or in combination. Apart from clinical evaluation, nocturnal oximetry and capnography monitoring and/or poly(somno)graphy (P(S)G) are usually carried out during the follow-up visits to monitor the delivered pressure, leaks, residual respiratory events and synchrony between the patient and the ventilator. Built-in software data of CPAP/NIV devices can be used to assess the adherence of treatment, to monitor pressure efficiency, leaks, asynchronies, and to estimate the presence of residual respiratory events under CPAP/NIV if P(S)G is not available or in alternance with P(S)G. The possibility of CPAP/NIV weaning should be assessed on a regular basis, but no criteria for the timing and procedures have been validated. Weaning timing depends on the clinical condition that justified CPAP/NIV initiation, spontaneous improvement with growth, and the possibility and efficacy of various upper airway, maxillofacial and/or neurosurgical procedures. Weaning may be allowed in case of the disappearance of nocturnal and daytime symptoms of sleep-disordered breathing (SDB) after several nights without CPAP/NIV and the objective correction of SDB on a P(S)G. But no parameters are defined. In any case, a long term follow-up is necessary to ascertain the weaning success. Large prospective studies, together with international and national guidelines, are required in order to build evidence for standardizing practice for the follow-up and weaning of CPAP/NIV in children.

## Introduction

Children receiving home noninvasive mechanical ventilation, by continuous positive airway pressure (CPAP) or noninvasive ventilation (NIV), should be followed with regular visits in order to check the tolerance and efficacy of the treatment. The follow-up strategy depends on various factors, such as the underlying disorder, the medical stability, the age of the child, and socio-economic factors, with no clear recommendations concerning follow-up visits and evaluation of efficiency of ventilation for children (1). The follow-up may consist of an overnight hospital stay, an outpatient visit, a home visit, a visit via telemedicine, alone or in combination. The evaluation of the respiratory support efficacy relies on sleep studies (poly(somno)graphy, P(S)G), nocturnal pulse oximetry (SpO_2_) and capnography (CO_2_) monitoring and/or respiratory support devices data with direct access of the data or via telemonitoring.

Follow-up may also help to identify patient's improvements that may prompt a weaning attempt from home CPAP/NIV. Indeed, as CPAP/NIV is usually initiated in children presenting with various medical conditions with different clinical evolutions and/or potential strategies (surgery, etc.), the need to reassess the utility for this ongoing treatment is crucial, as many children may be weaned from their respiratory support. Here again, no recommendations are available to guide the weaning process that is therefore based on clinical practice (2).

This review describes the available strategies based on the experience of worldwide centers or consensus papers, as evidenced-based guidelines for the follow-up and weaning of children receiving CPAP/NIV are lacking.

## Follow-Up

### Visits

Prior to hospital discharge, the patients and/or families must have received adequate education and training for the use of the home noninvasive respiratory support (3). Following hospital discharge, the Canadian Thoracic Society recommends that “the first visit and the frequency of subsequent visits should be tailored to the child and family's need” (1), based on a consensus for pediatric home ventilation (4). They suggested, based on a review of the literature, that the first visit should occur within the first month and no later than 3 months after home discharge (1). Following the first visit, the number and timing of the subsequent follow-up visits will depend upon the condition of the child and the evolution of the disease. Reassessment is recommended at least every 6–12 months (5, 6), even though more frequent follow-up visits may be necessary in many young children with additional unscheduled visits.

#### In-Hospital and Outpatient Clinic Visits

No studies have evaluated the optimal frequency and type of follow-up visits and monitoring resources for children using CPAP or NIV. Clinic follow-up visits may occur monthly to annually, depending on the patient's condition and underlying disease (3, 7).

In-hospital visits may be reserved for follow-up sleep studies, together with the assessment of the patient's ventilator use and the review of training and education of the patient and family when necessary (3, 8–10). According to our experience in children treated by CPAP, in-hospital P(S)G on CPAP may be limited to children with persistent symptoms of obstructive sleep apnea despite good objective adherence to treatment or when the nocturnal gas exchange and data obtained from built-in monitoring devices are abnormal or non-interpretable (11). Concerning patients treated by NIV, in-hospital P(S)G should be more systematic (10, 12), even though nocturnal gas exchange and data obtained from built-in monitoring devices (with breath-by-breath analysis of built-in software) may be sufficient for follow-up in a majority of patients (13).

**Figure 1:**
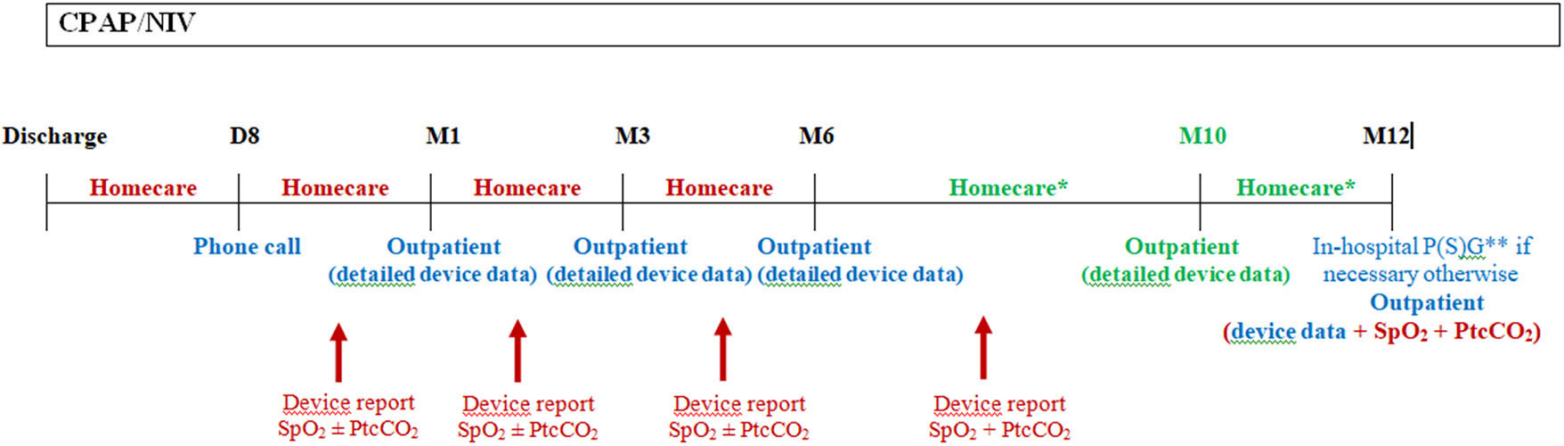
Schema of the first year follow-up in our center following hospital discharge. Following hospital discharge, a phone call to the family is done after 1 week by our staff (usually by a nurse specialized in therapeutic education) to ascertain the correct comprehension of the treatment and deal with the potential problems with the device and interface. A home visit is also performed by the homecare provider. A CPAP/NIV device data report and nocturnal gas exchange are done by the homecare provider and send to the hospital staff prior to the outpatient visit at 1 month following discharge. The same is usually repeated at 3, 6, and 10 (for NIV patients) months following discharge. In patients with CPAP, nocturnal gas exchange monitoring is not necessary at each homecare provider visit if the patient had no residual respiratory events under CPAP with a correction of gas exchange at 1 month follow-up. Moreover, transcutaneous carbon dioxide monitoring is not mandatory in patients under CPAP if they were not hypercapnic at the 1 month control. In this case, only nocturnal SpO_2_ may be performed at home if necessary for the following visits. Supplementary homecare provider or outpatients visits may be necessary in severe patients or in families with difficulties. And in-hospital admission may be necessary in case of clinical deterioration or if CPAP/NIV adjustments needs to be done with P(S)G. This schema is applicable for children who do not require surgery within the first year or who are not weanable, otherwise CPAP/NIV should be stopped and P(S)G and/or nocturnal gas exchange should be scheduled after the requested time following surgery or CPAP/NIV discontinuation due to spontaneous improvement. D, day; M, month; Homecare, homecare provider visit; Outpatient, outpatient hospital visit; SpO_2_, pulse oximetry; PtcCO_2_, transcutaneous carbon dioxide; P(S)G, poly(somno)graphy. In red: homecare services; In blue: hospital services; In green: only for patients with NIV. * At least 1 visit between M6 and M12, for patients with CPAP. ** Ideally P(S)G for patients with NIV.

Outpatient clinic visits allow to check for the patient's clinical condition, the adherence to treatment and the review of the ventilator data and needs for parents and child's retraining and education (8, 9, 11, 14, 15). They are feasible in patients treated by CPAP (11), but also by NIV (7). However, outpatient clinic visits rely on the availability of homecare providers or dedicated healthcare staff to assess the patient at home. Indeed, due to the limited time available during an outpatient clinic visit, assessments such as transcutaneous gas exchange may not be feasible and may not be representative of a nighttime monitoring. Therefore, a close collaboration with homecare providers is essential as they can do frequent home visits and send out the ventilator data and/or overnight recordings of gas exchange to the hospital team, prior to the outpatient clinic visit, which enables rapid adjustments of the settings when necessary, during the first weeks of treatment which are crucial for adherence, but also during follow-up (Figure 1).

#### Home Visits

Healthcare systems vary according to countries. Some centers have teams of professionals (mainly nurses, respiratory therapists but also physicians) who can perform home visits between hospital visits, while other centers collaborate with home healthcare providers who can visit the child and family (1, 9, 11, 14, 15). Several types of home service providers may exist with different medical functions (7). In a national survey in Canada, Rose et al. (7) reviewed the procedures or tests that were carried out during follow-up visits. They comprised regular ventilator checks, ventilator compliance/adherence assessment, overnight or daytime oximetry, overnight transcutaneous CO_2_ monitoring, spirometry and P(S)G, with an exclusive management at home in 53% of the cases. However, it is not clear in this review if tests such as spirometry or PSG were also carried out at home.

No published study compared hospital visits to home visits concerning follow-up strategies, probably because most patients may not be followed exclusively at home for their respiratory support treatment.

#### Telemedicine

Several studies reported the use and effectiveness of telemedicine for the follow-up care of children on home mechanical ventilation, with an increased interest in this strategy (7, 16–21). Telemedicine was first used to follow the children on home mechanical ventilation in the 1990s, and telemedicine is now largely implemented in many centers, to facilitate the patient's care and/or to limit the number of clinic visits or in-hospital admissions (19, 21). Studies showed a good acceptance by the patients and families, and by the paramedical and medical teams (20, 21). The telemedicine procedure associated real-time discussions with the patient, the family and professionals, the transmission of clinical data, SpO_2_ and transcutaneous CO_2_ monitoring, spirometry and ventilator data (16, 17, 21). However, as stated by Chuo et al. (16), “There is a “booming use” of telehealth without a corresponding “booming understanding” of its impact, advantages, and limitations.” Moreover, telemedicine may also cause an increased number of hospitalizations, oupatients visits, telemedicine consultations or home care visits (16), therefore “a more comprehensive cost benefit analysis is needed” together with legal clarity as legal problems associated with telemonitoring remain controversial. Indeed, although well accepted by patients, families and medical teams, the use of telemedicine needs further investigations as it is not clear if it actually decreases cost or improves outcome (1). The ERS statement on telemonitoring of ventilator-dependent patients concluded that “despite the hopes in telemonitoring as a means to face these problems, much more research is needed before considering telemonitoring a real improvement in the management of these patients” (17).

### Monitoring

Effectiveness of CPAP/NIV should be assessed on a regular basis. Monitoring should include ideally a P(S)G or nocturnal SpO_2_ and capnography recording, if P(S)G is not available. The monitoring should be performed every year at a minimum. Moreover, the respiratory support device built-in software data should be gathered at each clinic or home visit to assess the efficiency of ventilation, the adherence to the treatment, as well as the history of alarms and other events. Ideally, the same data used during in-hospital or outpatient visits to monitor the efficiency of ventilation and the clinical condition of the patient should be available during the home visit. Figure 1 shows our clinical practice for the first year follow-up of patients with CPAP/NIV, with the different monitoring resources available in our center.

#### Poly(somno)graphy (P(S)G)

International guidelines recommend to periodically perform PSG to revaluate children on CPAP (22). In children under NIV, with diseases such as neuromuscular disorders and alveolar hypoventilation syndromes, they proposed that a periodic reassessment with PSG and CO_2_ monitoring should be scheduled according to the child's growth rate and degree of clinical stability, but it should be at least annual (22, 23). Recent guidelines also recommended performing PSG to assess the effectiveness of ventilation every 6 month or every year (1). However, little evidence is available in the literature. Indeed, only two studies documented the usefulness of follow-up respiratory support titration sleep studies in children. Tan et al. (24) reported changes in the respiratory support settings in 66% of children and concluded that titration PSGs “lead to important management changes in children on respiratory support.” In the second study 53% of the PSG studies led to a modification in ventilator settings (25). The authors concluded that their study suggests that consequent changes in respiratory support settings allow an improvement in symptoms and that their findings support the current guidelines. PSG may also be valuable as it also allows recording of the mask pressure in order to monitor the delivered pressure, but also detects leaks and patient-ventilator synchrony (3), as well as identifying and characterizing residual respiratory events (26, 27). Whether P(S)G should be done only during in-hospital overnight or also at home (23) is not clear, because of the lack of studies reporting the use and feasibility of P(S)G at home in children receiving noninvasive respiratory support (28, 29).

However, the review of the experience of large centers highlights the fact that not all the centers are doing PSG, and that even when possible, the sleep studies are not done on a regular basis in all patients (1). This can be explained in part by the limited accessibility to sleep centers or to the confidence in home SpO_2_ and CO_2_ monitoring and respiratory support device data.

#### Nocturnal Pulse Oximetry and Capnography Monitoring

Guidelines usually recommend that, at minimum, overnight SpO_2_ and CO_2_ recordings should be available for the assessment of effectiveness of ventilation (23). However, as for respiratory support initiation, criteria to ascertain efficient ventilation are not clearly defined (30). Overnight CO_2_ monitoring is essential as normal SpO_2_ is not necessarily indicative of effective ventilation (3, 31, 32). The timing of follow-up nocturnal gas exchange is not defined, however the monitoring should at least be repeated if the ventilator settings are changed, or following an acute respiratory exacerbation. Overnight SpO_2_ with PtcCO_2_ recording should be performed more frequently in patients with NIV as numerous asymptomatic patients remain hypercapnic during sleep despite NIV use, or due to disease progression and needs for settings adjustments (Figure 1).

Regular assessment at home of the efficiency of ventilatory support and gas exchange recording appears to be consensual (1, 23). Indeed, studies reported feasibility and positive impact of home assessments because the child and family are in their usual environment (9, 23, 33). Either homecare providers or institutional care staffs are in charge of such monitoring, but the parents are also able to collaborate quite well (33).

#### Analysis of Data Obtained From Built in Monitoring Devices

Data from noninvasive respiratory support devices are widely used in clinical practice as important improvements have been made on the built-in software of CPAP/NIV devices (34, 35). Data such as adherence and effectiveness of ventilatory therapy are available, even though the type and number of available data vary according to the device (36). However, caution must be taken about interpretation of those data and technical specificities of the devices, as they are designed for adult patients and not for the pediatric population. Indeed, all the devices have manufacturer recommendations concerning the minimal weight of the patient. The device may therefore not be able to detect the breathing pattern of a child with a body weight below the minimal recommended weight, and thus may underestimate the real ventilator use by the patient and display erroneous data.

**Figure 2:**
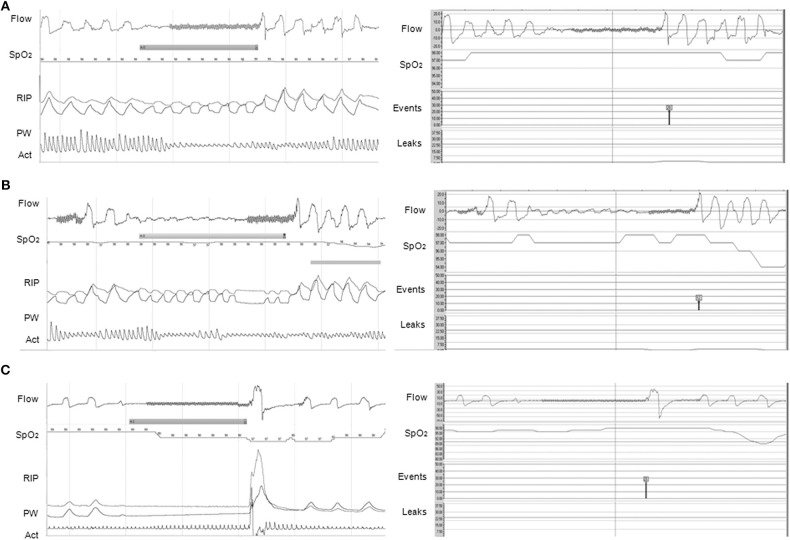
Agreements between PG (left panel) and CPAP (right panel) software for the events detection. **(A)** Obstructive apnea without 3% OD correctly scored by CPAP software (red symbol); **(B)** Hypopnea with 3% OD on PG. The event was scored as obstructive apnea on the CPAP because of the end of the event (red symbol); **(C)** Central apnea of >20 s but no 3% OD, possibly due to an artifact on SpO_2_ (note the artifacted pulse wave plethysmography), on PG compared to a central apnea of >20 s with 3% OD on CPAP (black symbol). On PG, Flow is expressed in L/min; SpO_2_ in %; RIP, respiratory inductance plethysmography; PW, pulse wave plethysmography; Act, actimetry. On CPAP software (ResScan^TM^, ResMed): Flow is expressed in L/min; SpO_2_ in %; Events represent the type of automatic scored event; Leaks are expressed in L/min. The continuous line represents the threshold of excessive leaks (24 L/min). CPAP devices were AirSense 10 **(A,B)** and S9 Elite **(C)**. (1 min period-window) From Khirani et al. (37).

**Figure 3:**
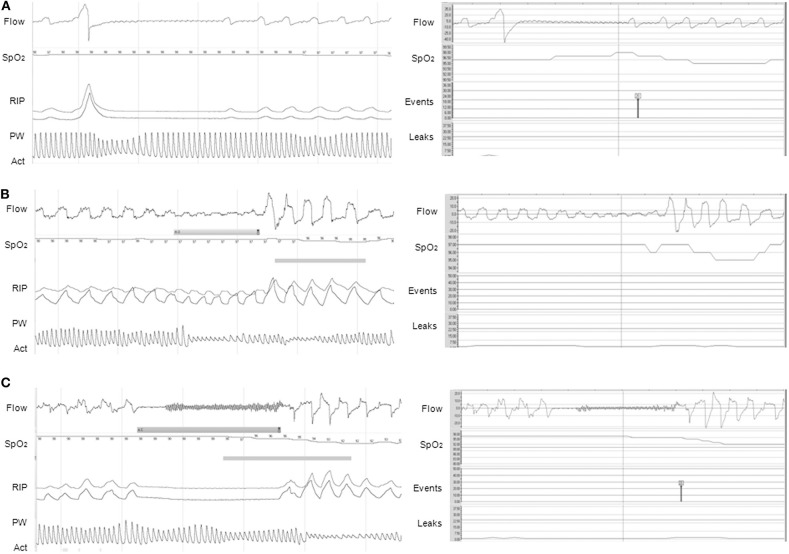
Discrepancies between PG (left panel) and CPAP (right panel) for the events detection. **(A)** Apnea preceded by a sigh, not scored on PG but automatically scored on CPAP (black symbol). Note the presence of a 3% OD on CPAP, not present on PG; **(B)** Obstructive hypopnea with consecutive 3% OD scored on PG but not scored automatically on CPAP; **(C)** Apnea with 3% OD scored as central on PG because of the absence of inspiratory effort, but falsely characterized as obstructive on CPAP (red symbol) (3% OD also present on CPAP). On PG, Flow is expressed in L/min; SpO_2_ in %; RIP, respiratory inductance plethysmography; PW, pulse wave plethysmography; Act, actimetry. On CPAP software (ResScan^TM^, ResMed): Flow is expressed in L/min; SpO_2_ in %; Events represent the type of automatic scored event; Leaks are expressed in L/min. The continuous line represents the threshold of excessive leaks (24 L/min). CPAP devices were S9 VPAP ST **(A)** and AirSense 10 **(B,C)**. (1 min period-window) From Khirani et al. (37).

Concerning CPAP or autoCPAP, data such as adherence, unintentional/intentional leaks, ventilation (tidal volume, minute ventilation, respiratory rate), pressure level, and residual respiratory events may be available. Breath-by-breath airflow and pressure curves can be downloaded from the majority of CPAP devices, allowing a more accurate overview of nighttime use and breathing pattern, ideally with the display of SpO_2_ directly in the software, and potential adjustments of the settings (Figure 2) (37). However, caution should be applied when reviewing the residual respiratory events and the apnea-hypopnea index data, as the algorithms for residual respiratory events on CPAP/NIV devices are based on adult criteria and do not follow the AASM scoring (Figure 3) (37, 38).

Concerning NIV, here again adherence, leaks, ventilation and residual respiratory events may be reviewed. Additional data such as patient-ventilator asynchronies may also be identified on the breath-by-breath data review (34). In many instances, residual respiratory events and patient-ventilator asynchronies can be managed by adjusting the ventilator settings and reviewing the ventilator data without the need of P(S)G, but in some cases P(S)G may be necessary (35).

Once again, no timing has been defined for the assessment of device data. However, the access to the respiratory support device data is becoming easier, as daily wireless transfer of data is now possible from many devices (36). This not only allows the patient to review their own therapy data, but also the homecare provider and healthcare staff to access remote monitoring of cloud-based data. The data can also be manually downloaded directly from the device during the in-hospital or outpatient visits. More detailed data such as breath-by-breath airflow and pressure curves from the ventilator are not still available through telemedicine. Availability of those data would allow early detection of changes, prompting rapid intervention and, potentially, prevention of hospital admission or prediction of respiratory exacerbations, even though data trends may be enough (35, 39). Indeed, telemonitoring of adherence is reliable—provided that the child's airflow is correctly detected by the device—and clinically useful as a good adherence to CPAP/NIV contributes to the improvement of clinical outcome. Irregular use of CPAP/NIV or increased use may indicate the necessity to review the patient for familial barriers to adherence or clinical course (poor tolerance, poor acceptance by the child and/or family, clinical deterioration, clinical improvement that may prompt weaning). Telemonitoring of leaks may be very helpful, as leaks may cause discomfort, poor adherence, patient-ventilator asynchrony and suboptimal efficiency of the ventilation to correct respiratory events, which should prompt intervention at home for adjustments and education. Telemonitoring of other data such as respiratory rate to predict respiratory exacerbation or clinical deterioration needs further research (39).

Follow-up review of the device data should be accompanied by at least SpO_2_ monitoring (at best SpO_2_ and transcutaneous CO_2_, that can now both be connected directly to some respiratory support devices). The efficacy of adjustments following leaks correction, correction of residual respiratory events or asynchronies should be ascertained by the normalization of nocturnal gas exchange (35). Future studies should focus on the interest of the combination of nocturnal gas exchange and built-in software data vs. PSG for the follow-up of CPAP/NIV.

## Weaning

The possibility of CPAP/NIV weaning should be assessed on a regular basis, but no criteria for the timing and procedures have been defined. Indeed, according to the disease, clinical evolution, and surgery strategy, many children, mostly with obstructive sleep apneas, may be weaned from their respiratory support (2, 8–10, 40, 41). P(S)G should be performed to confirm CPAP/NIV weaning, however when P(S)G is not feasible or available, the lack of sleep-disordered breathing symptoms and normal nocturnal gas exchange during sleep without CPAP/NIV may be a valuable alternative (9, 41). As there are neither recommendations nor guidelines for weaning children from ventilator support, in our clinical practice which concerns mainly patients with complex, genetic and rare diseases, a weaning trial is considered when four major criteria are fulfilled with at least two minor criteria (Table 1) (41). In clinical practice, however, the situation is not always so clear-cut and individual particularities should be considered such as the age of the patient, his/her pathology, the tolerance/acceptance and the subjective benefit of CPAP/NIV, and its potential side effects such as skin injury or facial deformity.

**Table 1:** Proposed criteria for a weaning attempt from continuous positive airway pressure (CPAP) or noninvasive ventilation (NIV).

Major criteria	•disappearance of nocturnal and daytime symptoms of sleep-disordered breathing after several nights without CPAP/NIV •(snoring, sweating, arousals, labored breathing, change in behavior or attention) •percentage of recording time spent with a SpO_2_ ≤ 90% < 2% •percentage of recording time spent with a PtcCO_2_ ≥ 50 mmHg < 2% •obstructive apnea-hypopnea index < 10 events/h on a poly(somno)graphy
Minor criteria	•minimal SpO_2_ > 90% •maximal PtcCO_2_ < 50 mmHg •oxygen desaturation index ≤ 1.4 events/h

*Adapted from Mastouri et al. (1)*.

*SpO_2_, pulse oximetry; PtcCO_2_, transcutaneous carbon dioxide*.

Due to the long lasting effects of CPAP/NIV, a delay of at least 2 weeks is recommended without respiratory support prior to a sleep study during spontaneous breathing (41). Indeed, several mechanisms may account for this prolonged benefit, such as an attenuation of mucosal edema, a reduction of sleep fragmentation which is known to worsen airway collapsibility, an improvement of the hypercapnic ventilatory response, and an increase in upper airway muscle tone. In patients with abnormalities of the upper airways, corrective surgery may cure obstructive sleep apneas. A minimal delay corresponding to the timing associated with the maximal benefit, lasting ~2–6 months according to the type of surgery, is recommended prior to performing a sleep study during spontaneous breathing. Finally, a progressive reduction of CPAP or NIV tolerance or compliance, in the absence of any problems, may be due to a spontaneous improvement of sleep disordered breathing, as it can be observed in infants with Pierre Robin syndrome or laryngomalacia (41). A long term follow-up is mandatory following CPAP/NIV withdrawal in children with associated disorders, as a relapse of obstructive sleep apnea may occur, particularly in syndromic children (41).

Concerning the children who discontinued CPAP/NIV due to poor tolerance or acceptance, alternative strategies may be proposed in selected patients (see the associated review in this series on “Which options when NIV fails”). Strategies such as intensive psychological and play specialist support (10) or medical hypnosis (42) can also be tested in patients who do not tolerate any interface or having other difficulties.

## Conclusions

Whether the evaluation of the effectiveness of home CPAP/NIV is performed at home or in-hospital, the assessments need to be done on a regular basis, ideally using the same monitoring resources. Due to the absence of evidence-based guidelines, follow-up strategies vary according to clinical practice but should aim to move toward building evidence for standardizing practice. Multicenter studies, together with international and national guidelines, are required to build evidence for standardizing practice for the follow-up and weaning of CPAP/NIV in children.

## Author Contributions

SK is the main author of the manuscript. BF, AA, LG, AL, and TT contributed to the management of the children followed in our center and the writing of the manuscript. All the authors approved the final version of the manuscript. All authors contributed to the article and approved the submitted version.

## Conflict of Interest

The authors declare that the research was conducted in the absence of any commercial or financial relationships that could be construed as a potential conflict of interest.
